# Comparative genomics of obligate predatory bacteria belonging to phylum *Bdellovibrionota* highlights distribution and predicted functions of lineage-specific protein families

**DOI:** 10.1128/msphere.00680-24

**Published:** 2024-11-13

**Authors:** Sidney C. Davis, Joseph Cerra, Laura E. Williams

**Affiliations:** 1 Department of Biology, Providence College, Providence, Rhode Island, USA; Clemson University, Clemson, South Carolina, USA

**Keywords:** predatory bacteria, genome evolution, pangenome

## Abstract

Comparative genomics of predatory bacteria is important to understand their ecology and evolution and explore their potential to treat drug-resistant infections. We compared chromosomes of 18 obligate predators from phylum *Bdellovibrionota* (16 intraperiplasmic, two epibiotic) and 15 non-predatory bacteria. Phylogenetics of conserved single-copy genes and analysis of genome-wide average amino acid identity provide evidence for at least five *Bdellovibrio* species and support recent reclassifications of predatory taxa. To define shared and differential genome content, we grouped predicted protein sequences into gene clusters based on sequence similarity. Few gene clusters are shared by all 33 bacteria or all 18 predatory bacteria; however, we identified gene clusters conserved within lineages, such as intraperiplasmic *Bdellovibrio*, and not found in other bacteria. Many of these are predicted to function in cell envelope biogenesis, signal transduction, and other roles important for predatory lifestyles. Among intraperiplasmic *Bdellovibrio*, we detected high abundance of gene clusters predicted to encode transglycosylases, endopeptidases, and lysozymes, and we identified six gene clusters (amidase, L,D-transpeptidase, four transglycosylases) with evidence of recent gene duplication and gene family expansion. Focusing on peptidoglycan metabolism, we defined a suite of gene clusters that include peptidoglycan-degrading and -modifying enzymes and occur only in predatory bacteria, suggesting these proteins may have evolved activities specific to predation. Our analyses highlight key genome content differences between obligate predatory bacteria and non-predatory relatives and identify gene clusters that may encode enzymes adapted to predatory lifestyles. These lineage-specific proteins are strong candidates for functional characterization to clarify their role in predation.

**IMPORTANCE:**

Evolution of predation as a bacterial lifestyle involves selective pressure on and adaptation of enzymes that contribute to killing and digestion of prey bacteria, in some cases from within the prey itself. Such enzymes are a hallmark of obligate predatory bacteria belonging to phylum *Bdellovibrionota*, which includes the well-studied predator *Bdellovibrio*. By comparing protein sequences of obligate predatory bacteria and their non-predatory relatives, we define key genome content differences that distinguish bacterial predators and identify lineage-specific enzymes that may have evolved unique activities due to selective pressures related to a predatory lifestyle. In addition to providing insights into the ecology and evolution of predatory bacteria, comparative genomics studies, like this, can inform efforts to develop predatory bacteria and/or their enzymes as potential biocontrol agents to combat drug-resistant bacterial infections.

## INTRODUCTION

Most bacteria experience predation as the unfortunate prey of protists and other creatures; however, a few bacterial species have evolved to hunt, attack, and digest their prokaryotic compatriots. Phylogenetic and genomic analyses point to independent evolution of predatory lifestyles multiple times across the bacterial tree of life, with predatory bacteria identified in a wide range of phyla (1). Facultative predatory bacteria, such as *Myxococcus*, are capable of killing other bacteria to fuel their growth and reproduction, but they can also acquire nutrients via other means, including degradation of dead organisms. By contrast, *Bdellovibrio* are considered obligate predators based on experimental evidence from the most well-studied representatives, which shows that *Bdellovibrio* depend on killing and consuming prey bacteria for their survival, growth, and reproduction. Here, we analyze evolutionary relationships, genome relatedness, and genome content variation among obligate predatory bacteria to better understand the diversity, distribution, and predicted function of protein families in these taxa.

Sequencing and annotation of *Bdellovibrio bacteriovorus* HD100 in 2004 provided the first obligate predatory bacteria genome and established a foundation for comparative genomics of predatory species (2). Completion of additional genomes and targeted sequencing of phylogenetically informative genes, such as the 16S rRNA gene, contributed to a broad picture of genome variation and phylogenetic diversity among *Bdellovibrio* and closely related predatory bacteria (3–9). These data informed continuous re-evaluation and refinement of taxonomy, leading to a recent proposal to gather some obligate predatory genera, including *Bdellovibrio*, *Bacteriovorax*, and *Halobacteriovorax*, into a novel phylum named *Bdellovibrionota* (10). Most described predatory strains in this phylum evolved a strategy of intraperiplasmic predation, in which they invade the periplasm of Gram-negative prey, then digest the prey cell’s cytoplasmic contents to grow and reproduce. However, a few strains in this phylum, such as *Pseudobdellovibrio exovorus* JSS (11), are not known to invade the prey periplasm and instead consume prey cytoplasmic contents while remaining attached to the outside of the prey cell, a strategy referred to as epibiotic predation.

The requirements of a predatory lifestyle, whether intraperiplasmic or epibiotic, result in unique selective pressures and thereby affect molecular evolution of proteins encoded by the genomes of predatory bacteria. Successful predation relies on many different lytic enzymes to access prey cell cytoplasm and digest its contents. Intraperiplasmic predators are additionally faced with the challenge of controlled modification and degradation of prey cell peptidoglycan to enable invasion of the prey periplasm, maintenance of a stable environment within the prey cell during growth and reproduction, and lysis of dead prey cell envelope to release progeny. Annotation of the HD100 genome suggested that predatory bacteria possess an expanded suite of lytic enzymes compared to non-predatory relatives (2), and recent characterization of some of these enzymes revealed novel activities related to predation, such as deacetylation of prey peptidoglycan to mark it for eventual degradation by specialized lysozymes (12–14). Such predation-adapted proteins are key features that distinguish predatory bacteria from their non-predatory relatives and contribute to the evolution of a predatory lifestyle.

To identify lineage-specific protein families, define their distribution, and determine their predicted functions, we leveraged the publicly available complete genome data set for *Bdellovibrionota*, which has expanded significantly over the past 20 years due to the efforts of multiple research groups. The comparative genomics analyses presented here provide insight into genome content variation characteristic of obligate predatory bacteria and highlight lytic enzyme families that may encode novel functions specifically adapted to predation. These enzymes are strong candidates for functional characterization and possibly development as tools for biotechnological applications or clinical applications to treat antibiotic-resistant Gram-negative bacterial infections.

## RESULTS

### Phylogenetic and AAI analyses support recently proposed reclassifications of predatory bacteria and highlight predatory species diversity within *Bdellovibrionota*

**Fig 1:**
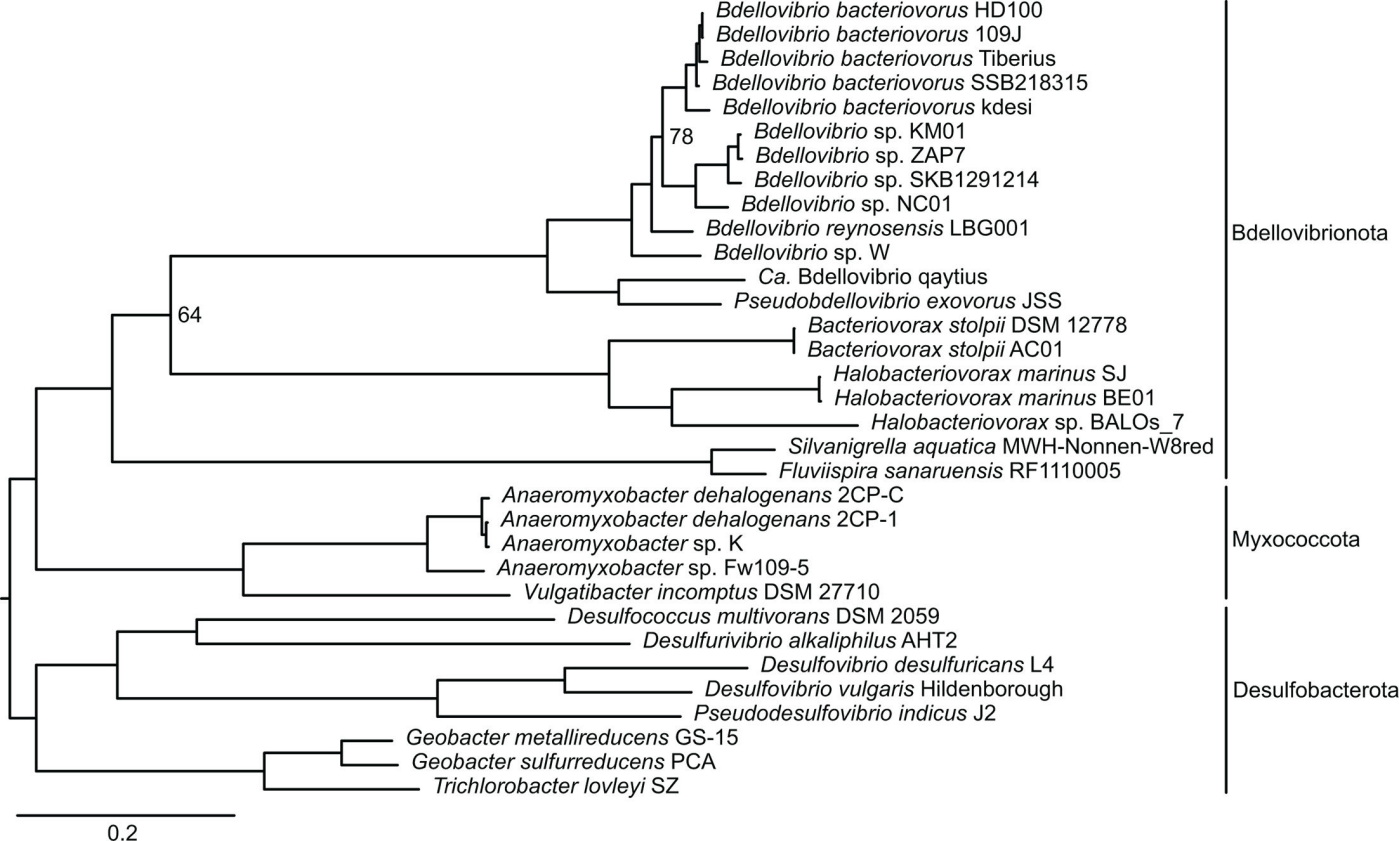
Evolutionary relationships of predatory *Bdellovibrionota* and related non-predatory bacteria. We inferred a maximum likelihood phylogenetic tree from 44 conserved single-copy gene clusters. Strains belonging to *Bdellovibrio*, *Pseudobdellovibrio*, *Bacteriovorax*, and *Halobacteriovorax* are obligate predators, whereas all other strains on the tree are not currently known to exhibit predatory behavior. Phylum classifications based on reference (10) are indicated by vertical lines to the right of taxon labels. We manually rooted the tree on the branch leading to the *Desulfobacterota* clade. All nodes had ≥97% support via ultrafast bootstrap approximation with the exception of two nodes, for which support values are shown on the tree.

**Fig 2:**
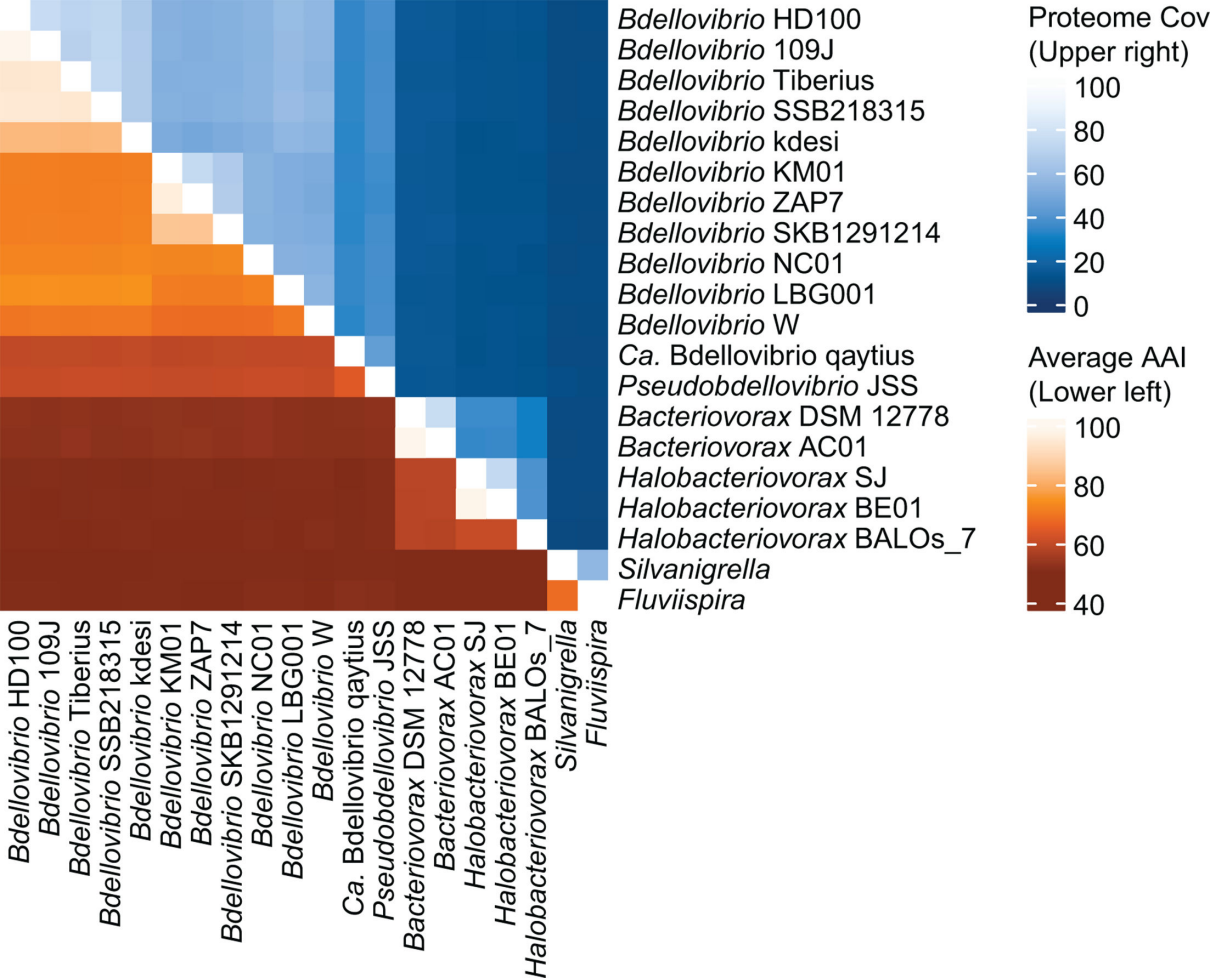
Average amino acid identity and proteome coverage for 20 *Bdellovibrionota* strains. The lower left triangle of the heatmap uses a red gradient to show average amino acid identity, which is an overall estimate of the similarity of bidirectional best hit protein sequences for a given pair of chromosomes. The upper right triangle of the heatmap uses a blue gradient to show proteome coverage, which is the proportion of protein sequences from a given pair of chromosomes that are bidirectional best hits. Strains are ordered in the heatmap to match the phylogenetic tree in Fig. 1.

Our data set comprises 33 complete chromosome sequences from 18 obligate predatory bacteria and 15 non-predatory bacteria belonging to phyla *Bdellovibrionota*, *Myxococcota*, and *Desulfobacterota* (Table 1). To reconstruct evolutionary relationships among these bacteria, we identified single-copy genes conserved among all 33 chromosomes and inferred a maximum likelihood phylogenetic tree from the protein sequences (Fig. 1). To estimate genome relatedness among the 20 *Bdellovibrionota* strains (18 predatory and two non-predatory), we calculated average amino acid identity (AAI) and proteome coverage for all possible pairwise comparisons (Fig. 2).

**TABLE 1:** Chromosome sequences of predatory bacteria belonging to phylum *Bdellovibrionota* and related non-predatory bacteria

Family	Genus	Species	Strain	Chrom (Mb)	GenBank accession	Assembly accession
Predatory						
*Bdellovibrionaceae*	*Bdellovibrio*	*bacteriovorus*	HD100	3.783	NC_005363	GCF_000196175.1
			109J	3.830	NZ_CP007656	GCF_000691605.1
			kdesi^*a*^	3.344	NZ_CP102930	GCF_025558485.1
			SSB218315	3.770	NZ_CP020946	GCF_002208115.1
			Tiberius	3.989	NC_019567	GCF_000317895.1
		*reynosensis*	LBG001	3.582	NZ_CP093442	GCF_022814725.1
		sp.	KM01	3.961	NZ_CP058348	GCF_013752535.1
		sp.	NC01	3.976	NZ_CP030034	GCF_006874625.1
		sp.	SKB1291214	3.681	NZ_CP106855	GCF_002209355.2
		sp.	W^*a*^	3.007	CP002190	GCF_000525675.1
		sp.	ZAP7	4.124	NZ_CP030082	GCF_006874645.1
	*Ca.* Bdellovibrio	qaytius		3.349	CP025734	GCA_004011035.1
	*Pseudobdellovibrio*	*exovorus*	JSS	2.658	NC_020813	GCF_000348725.1
*Bacteriovoracaceae*	*Bacteriovorax*	*stolpii*	DSM 12778	3.810	NZ_CP025704	GCF_002872415.1
			AC01	3.881	NZ_CP030035	GCF_006874605.1
	*Halobacteriovorax*	*marinus*	SJ	3.436	NC_016620	GCF_000210915.2
			BE01	3.393	NZ_CP017414	GCF_002442875.1
		sp.	BALOs_7	3.153	NZ_CP027772	GCF_003612895.1
Non-predatory^*b*^						
*Silvanigrellaceae*	*Silvanigrella*	*aquatica*	MWH-Nonnen-W8red	3.342	NZ_CP017834	GCF_001907975.1
	*Fluviispira*	*sanaruensis*	RF1110005	3.520	NZ_AP019368	GCF_004295685.1
*Anaeromyxobacteraceae*	*Anaeromyxobacter*	*dehalogenans*	2 CP-C	5.013	NC_007760	GCF_000013385.1
			2 CP-1	5.029	NC_011891	GCF_000022145.1
		sp.	Fw109-5	5.278	NC_009675	GCF_000017505.1
		sp.	K	5.062	NC_011145	GCF_000020805.1
*Vulgatibacteraceae*	*Vulgatibacter*	*incomptus*	DSM 27710	4.351	NZ_CP012332	GCF_001263175.1
*Desulfococcaceae*	*Desulfococcus*	*multivorans*	DSM 2059	4.456	NZ_CP019913	GCF_002009335.2
*Desulfurivibrionaceae*	*Desulfurivibrio*	*alkaliphilus*	AHT 2	3.098	NC_014216	GCF_000092205.1
*Desulfovibrionaceae*	*Desulfovibrio*	*desulfuricans*	L4	3.544	NZ_CP072608	GCF_017815575.1
		*vulgaris*	Hildenborough	3.571	NC_002937	GCF_000195755.1
	*Pseudodesulfovibrio*	*indicus*	J2	3.967	NZ_CP014206	GCF_001563225.1
*Geobacteraceae*	*Geobacter*	*metallireducens*	GS-15	3.997	NC_007517	GCF_000012925.1
		*sulfurreducens*	PCA	3.814	NC_002939	GCF_000007985.2
*Pseudopelobacteraceae*	*Trichlorobacter*	*lovleyi*	SZ	3.918	NC_010814	GCF_000020385.1

^*a*^ Chromosomes of *Bdellovibrio* strains kdesi and W are smaller than average for this genus, and there are some indications these two genomes may not be complete (see Materials and Methods).

^*b*^ Based on a literature search as of December 2022. It is possible these bacteria have undiscovered predatory capabilities; however, they are not obligate predators.

On the phylogenetic tree (Fig. 1), the 33 bacteria cluster into three clades consistent with phylum reclassifications recently proposed by Waite and colleagues (10). Within phylum *Bdellovibrionota*, predatory bacteria belonging to *Bdellovibrio*, *Pseudobdellovibrio*, *Bacteriovorax*, and *Halobacteriovorax* cluster together and apart from non-predatory *Silvanigrella* and *Fluviispira*. Only a small proportion of protein sequences (7%–10%) are bidirectional best hits in pairwise comparisons of predatory and non-predatory bacteria within *Bdellovibrionota* (Fig. 2), and AAI of these pairwise comparisons are relatively low (49%–51%). This reflects the phylogenetic and evolutionary distance between predatory and non-predatory lineages in our data set.

Most of the obligate predatory bacteria in our data set use a predation strategy involving invasion of the prey’s periplasm; by contrast, JSS and qaytius are epibiotic predators and are not known to invade prey cells. On the phylogenetic tree, these two strains form a distinct lineage apart from the intraperiplasmic predators, which supports the recent proposal by Waite and colleagues (10) to reclassify *Bdellovibrio exovorus* JSS as a type strain within the new genus *Pseudobdellovibrio*. Comparing JSS and *Candidatus* (*Ca*.) Bdellovibrio qaytius protein sequences yielded 64% AAI with almost half of their proteomes identified as bidirectional best hits. This AAI value is just below a commonly applied cutoff (65%) for delineating bacterial genera (15). Given that AAI between qaytius and each of the intraperiplasmic *Bdellovibrio* never exceeds 60%, *Ca.* Bdellovibrio qaytius likely belongs in a different genus than *Bdellovibrio*, but whether it should be classified with JSS as *Pseudobdellovibrio* is not clear from these analyses.

Within *Bdellovibrio*, *B. bacteriovorus* HD100 and 109J are the most well-studied strains. Comparing their chromosomes, most of these two strains’ protein sequences (75%) are reciprocally matched, and these bidirectional best hits are highly similar with 99% AAI. Over the past few years, isolation and sequencing of *Bdellovibrio* strains, such as KM01 (16), SKB129121 (6), NC01 (7), and LBG001 (8), have expanded the diversity of publicly available *Bdellovibrio* genomes. Considering these four strains, examination of their positions on the phylogenetic tree (Fig. 1) and comparison of their proteomes to HD100 and each other (Fig. 2) suggest each strain likely represents a distinct species. Indeed, LBG001 was recently proposed as the type strain for novel species *Bdellovibrio reynosensis* (8). Overall, phylogenetic and AAI analyses suggest at least five *Bdellovibrio* species within our chromosome data set, emphasizing diversity and variation within this predatory genus.

### Pangenome analysis identifies few core and many singleton gene clusters

To analyze shared and differential genome content among bacteria in our data set, we used anvi’o (17, 18) to build three pangenomes: FULL, consisting of all 33 bacteria; PRED, consisting of 18 predatory bacteria; and BDELLO, consisting of 13 predatory bacteria belonging to the Family *Bdellovibrionaceae*. For all chromosomes in a pangenome, anvi’o translated DNA sequences from predicted open reading frames, then grouped amino acid sequences by similarity using the MCL clustering algorithm (19), thereby generating “gene clusters.” As expected, the total number of gene clusters varies with the MCL inflation parameter (Table 2), which defines cluster granularity. Lower parameter values yield fewer gene clusters, as more sequences are grouped together. Very coarse clusters may accurately recapitulate protein superfamilies, but these broad groupings will obscure potentially important molecular evolution among proteins within a superfamily. We elected to use the lowest inflation parameter value tested (mcl 1.4), which generates coarse gene clusters, but distinguishes among divergent proteins within a superfamily. With this approach, we aim to identify predatory bacteria proteins that have diverged substantially from homologous proteins in other lineages. Examining the resulting gene clusters, there were very few gene clusters conserved among all 33 bacteria (*n* = 57 in FULL) or among all 18 predatory bacteria (*n* = 152 in PRED). In addition, each pangenome has a high proportion of singleton gene clusters, which are comprised of protein sequences occurring in only one chromosome.

**TABLE 2:** Effect of MCL inflation parameter on number of gene clusters in pangenomes

	FULL			PRED			BDELLO		
	Total	Core^*a*^	Single^*b*^	Total	Core^*a*^	Single^*b*^	Total	Core^*a*^	Single^*b*^
mcl 1.4	49,264	57	30,209	19,733	152	9,130	12,266	818	6,832
mcl 2	49,596	46	30,216	19,881	138	9,130	12,368	802	6,832
mcl 6	50,757	37	30,355	20,502	126	9,158	12,544	784	5,800

^*a*^ Core gene clusters are those occurring in all chromosomes (*n* = 33 for FULL, *n* = 18 for PRED, *n* = 13 for BDELLO).

^*b*^ Singleton gene clusters are those occurring in only one chromosome.

**Fig 3:**
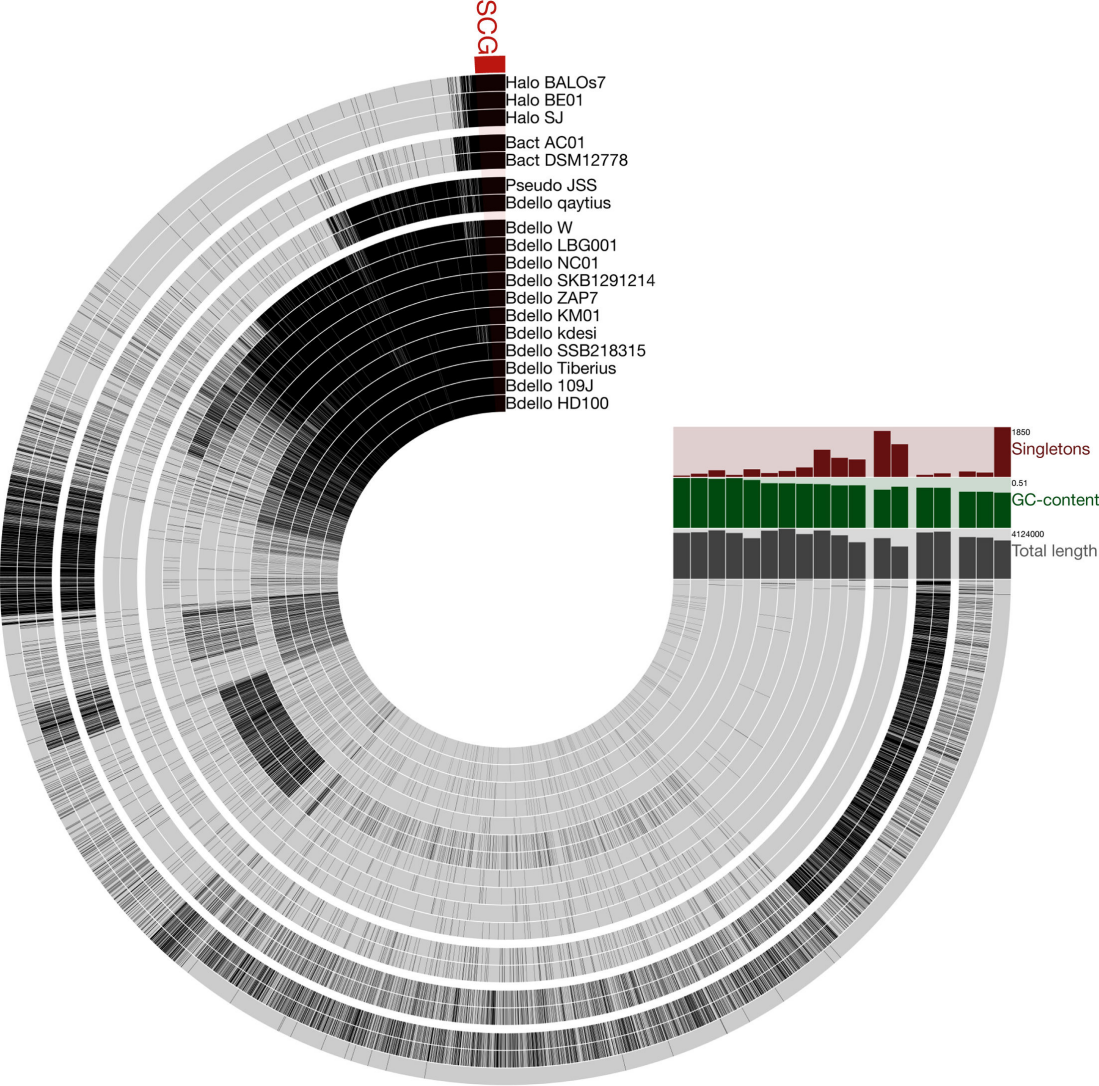
Pangenome of 18 predatory bacteria within *Bdellovibrionota*. Each track represents the chromosome of one strain. Tracks are ordered to match the phylogenetic tree in Fig. 1, with larger spaces between certain tracks to define clades more clearly. Within each track, black indicates presence of a gene cluster, whereas gray indicates absence. The distribution of a particular gene cluster can be determined by moving along the radius from the innermost to the outermost track. As an example, gene clusters occurring in all 18 predatory bacteria are grouped at the beginning of the tracks, with single-copy core gene clusters denoted by a red box labeled “SGC.” At the end of the tracks, three histogram panels show summary information about each chromosome. To simplify display of the pangenome, singleton gene clusters are summarized in one of the histogram panels and not represented in the tracks.

Figure 3 shows the PRED pangenome and highlights two recognized features about gene clusters as follows: (1) the number of shared gene clusters increases with smaller phylogenetic distances, and (2) the number of unique (singleton) gene clusters increases with larger phylogenetic distances. In the figure, gene clusters conserved among all 18 predatory bacteria are grouped at the beginning of the pangenome tracks. Between *Bacteriovoracaceae* (three *Halobacteriovorax* and two *Bacteriovorax*) and *Bdellovibrionaceae* (11 *Bdellovibrio* and two epibiotic strains), there are very few conserved gene clusters, but within *Bdellovibrionaceae*, the two epibiotic predatory bacteria (JSS and qaytius) share more gene clusters with *Bdellovibrio* strains. Regarding unique gene clusters, strains BALOs_7, JSS, and qaytius have the largest phylogenetic distances from other predatory strains (Fig. 1) and the highest number of unique gene clusters (see Singletons histogram panel in Fig. 3). By contrast, considering pairs of very similar strains with high AAI (Fig. 2), such as *Bdellovibrio bacteriovorus* HD100 and 109J, *Bacteriovorax stolpii* DSM 12778 and AC01, and *Halobacteriovorax marinus* SJ and BE01, these strains have very few singleton gene clusters, which is expected due to the high degree of genome relatedness within the pairs. Among *Bdellovibrio*, NC01 has the highest number of unique gene clusters (*n* = 1,011), even more than LBG001 (*n* = 717) and W (*n* = 649), which have similar or larger phylogenetic distances from other predatory strains. Unique gene clusters also comprise a higher proportion of total genes in NC01 (27%) compared to LBG001 (22%) and W (24%). This suggests that NC01 may have experienced a unique evolutionary trajectory with features that differentiate it from other *Bdellovibrio*.

### Gene clusters conserved among and specific to predatory lineages are associated with cell envelope biogenesis, signal transduction, and other functions important for predatory lifestyles

**Fig 4:**
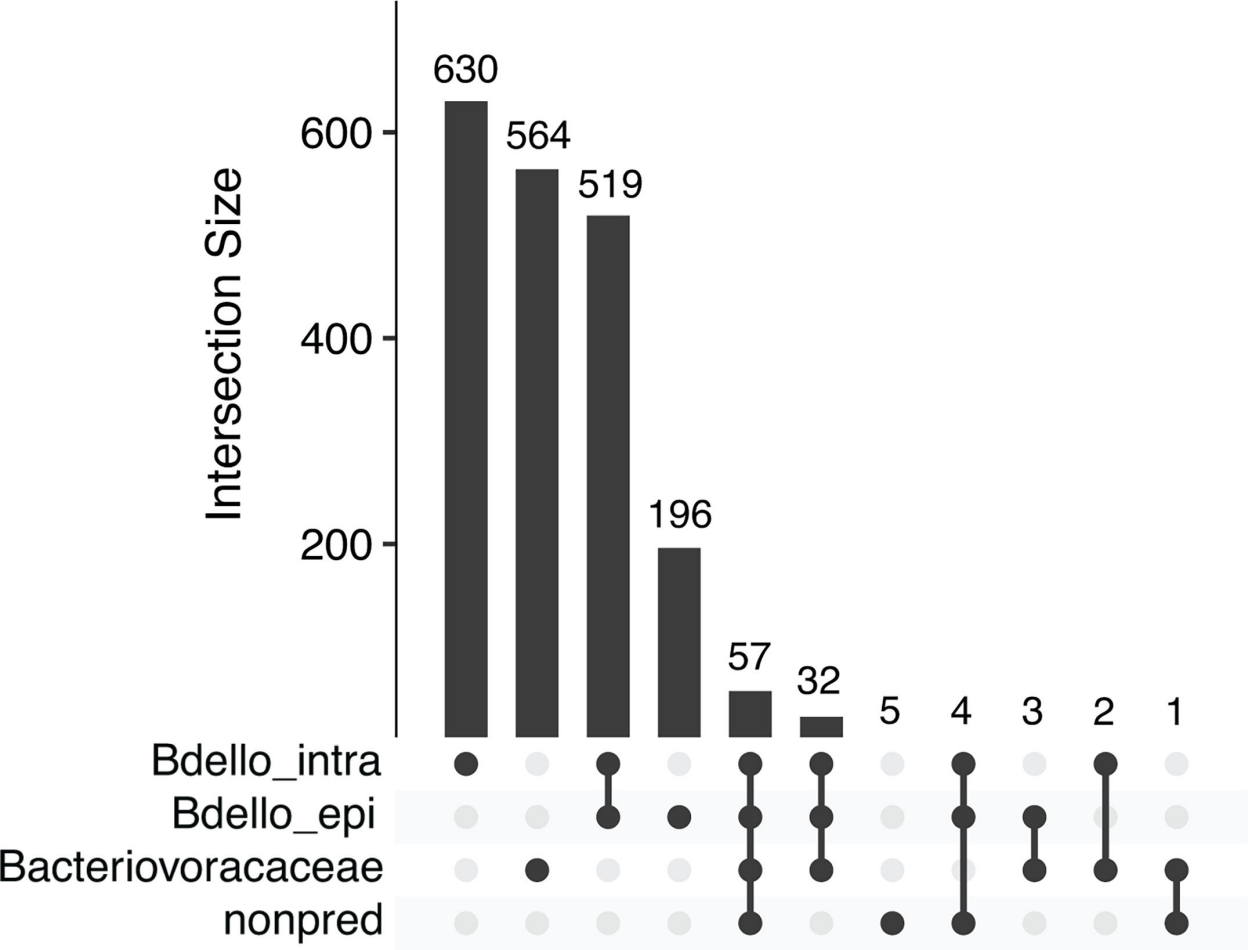
Gene clusters conserved among and specific to subsets of bacteria in the FULL pangenome. We grouped chromosomes into four subsets based on taxonomy and predation strategy (see text). Combinations of these subsets are indicated by black dots within the rows at the bottom of the figure, and the number of gene clusters occurring in those chromosomes are shown by the histogram. For example, the four black dots joined by a single line in the fifth column indicate a combination of all four subsets, which is the FULL pangenome with all 33 chromosomes, and the corresponding bar shows that 57 gene clusters are conserved among bacteria in this combination.

**Fig 5:**
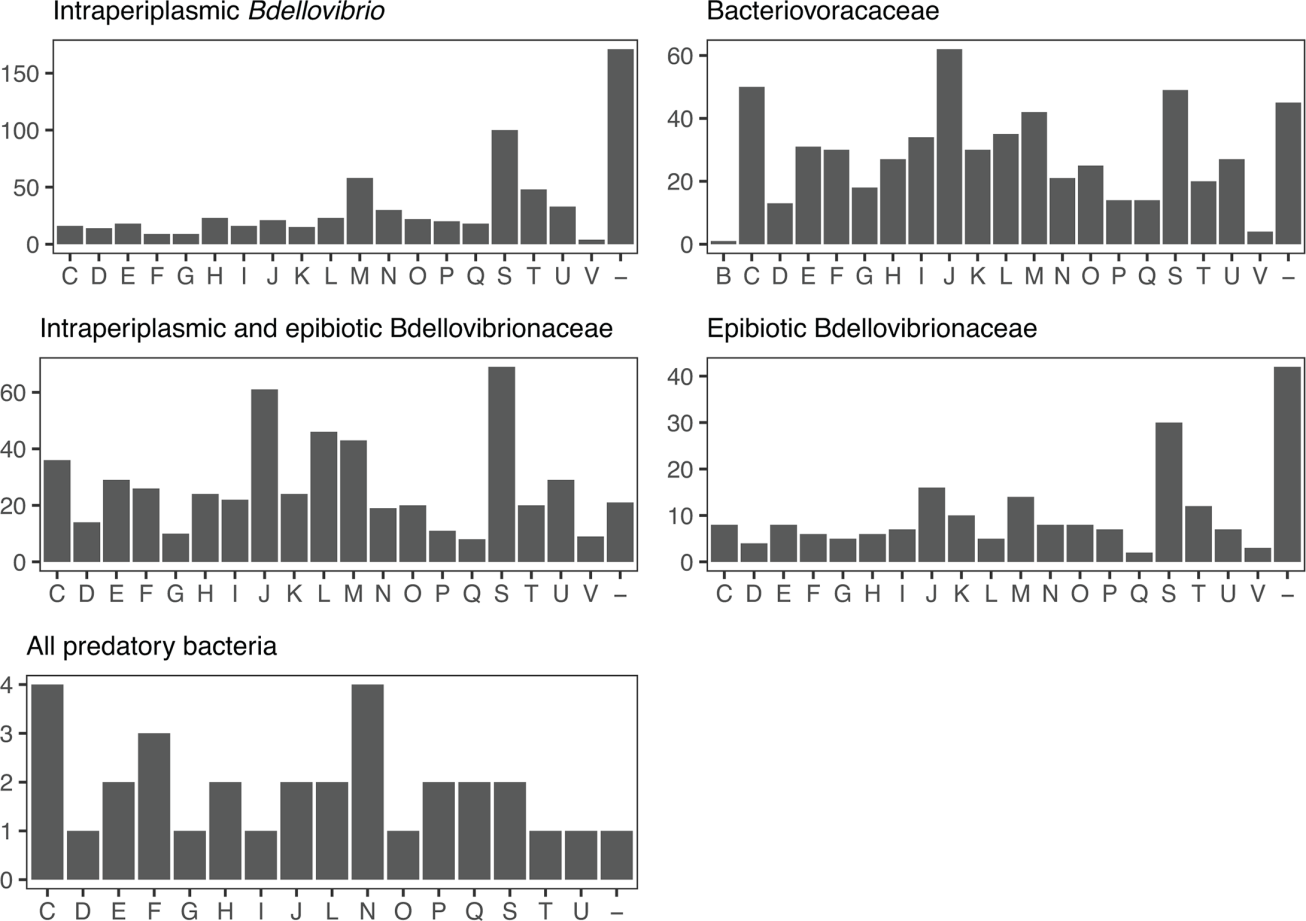
COG classifications of conserved and specific gene clusters within subsets of predatory bacteria. For the subsets defined in the UpSet plot in Fig. 4, we retrieved COG classifications for each gene cluster from functional annotation information in the FULL pangenome. Some gene clusters were classified into multiple COG categories and are therefore represented more than once in the histograms. COG categories are as follows: B = chromatin structure and dynamics; C = energy production and conversion; D = cell division and chromosome partitioning; E = amino acid metabolism and transport; F = nucleotide metabolism and transport; G = carbohydrate metabolism and transport; H = coenzyme metabolism and transport; I = lipid metabolism and transport; J = translation, ribosomal structure and biogenesis; K = transcription; L = replication, recombination, and repair; M = cell wall/membrane/envelope biogenesis; *N* = cell motility; O = posttranslational modification, protein turnover, chaperones; P = inorganic ion transport and metabolism; Q = secondary metabolites biosynthesis, transport, and catabolism; R = general functional prediction only; S = no functional prediction; T = signal transduction; U = intracellular trafficking, secretion, and vesicular transport; V = defense mechanisms. The dash indicates no COG classification.

The PRED pangenome shown in Fig. 3 displays gene cluster presence–absence data for individual predatory bacteria chromosomes. To better understand gene cluster distribution among our full chromosome data set, we grouped all 33 bacteria into four subsets based on taxonomy and predation strategy: Bdello_intra includes 11 intraperiplasmic *Bdellovibrio*, Bdello_epi includes two epibiotic predatory bacteria within *Bdellovibrionaceae, Bacteriovoracaceae* includes five *Bacteriovorax* and *Halobacteriovorax*, and nonpred includes 15 non-predatory bacteria. We then identified gene clusters shared by all chromosomes of one subset or combination of subsets and not found in any other chromosomes (Fig. 4). These gene clusters can be considered conserved among and specific to certain lineages of bacteria in our data set and may be associated with evolution of predatory lifestyle, including intraperiplasmic versus epibiotic strategies, or adaptation of predatory bacteria to different environments. To obtain general functional predictions for the gene clusters, we retrieved Clusters of Orthologous Genes (COG) classifications from annotation information in the FULL pangenome (Fig. 5).

Intraperiplasmic *Bdellovibrio* have the highest number of conserved and specific gene clusters (*n* = 630), followed by *Bacteriovoracaceae* (*n* = 564), the combined members of *Bdellovibrionaceae* (*n* = 519), and epibiotic *Bdellovibrionaceae* (*n* = 196). Many of these gene clusters have no COG functional information (either no COG classification or S, Fig. 5), with the highest proportions in intraperiplasmic *Bdellovibrio* (43%) and epibiotic predators (37%). Despite annotating each protein sequence with multiple tools during pangenome construction, additional annotation information for these gene clusters is sparse and primarily limited to broad identification of protein domains without detailed functional predictions. These gene clusters may encode divergent proteins with novel functions that are potentially important for predation.

Considering gene clusters with COG functional information, “cell envelope biogenesis” (M) is one of the top three COG categories for conserved and specific gene clusters from each of the lineages of predatory bacteria represented in Fig. 5. This is consistent with the importance of breakdown and modification of cell membranes and peptidoglycan in the predatory lifecycle. “Signal transduction” (T) is also a top three COG category for gene clusters conserved within and specific to the two separate lineages of *Bdellovibrionaceae*: Bdello_intra and Bdello_epi. These two different predation strategies may exert distinct selective pressures on proteins involved in signaling, which play key roles in events throughout predation (20, 21), and may result in molecular evolution and sequence divergence of signal transduction proteins beyond that correlated with phylogenetic distance. In addition, “intracellular trafficking, secretion, and vesicular transport” (U) is a top three COG category for intraperiplasmic *Bdellovibrio*. Movement of enzymes and compounds between predator and prey is essential for successful growth and reproduction via intraperiplasmic predation. Another prominent COG category is “translation, ribosomal structure, and biogenesis” (J), which is a top three COG category for Bdello_epi, combined members of *Bdellovibrionaceae*, and *Bacteriovoracaceae*.

Thirty-two gene clusters were conserved among all 18 predatory bacteria and absent from non-predatory bacteria in our data set. These may include proteins that evolved and diverged in response to selective pressures associated with a predatory lifestyle. Although no single COG category is represented substantially more than others, the top three COG categories are “energy production and conversion” (C), “cell motility” (N), and “nucleotide metabolism and transport” (F). The four gene clusters assigned to cell motility are annotated as flagellar proteins (HD100 locus tags Bd0611, Bd3403, Bd3395, Bd3407). Flagellar motility is considered important for both intraperiplasmic and epibiotic predatory bacteria to search for prey in certain environments (22).

Only two gene clusters are conserved among and specific to all intraperiplasmic predatory bacteria lineages (*Bdellovibrio*, *Bacteriovorax*, and *Halobacteriovorax*) in our data set: class I SAM-dependent methyltransferase (Bd1029) and NAD-dependent deacylase (Bd2785). Using BLASTP, we aligned Bd1029 and Bd2785 protein sequences from HD100 against the non-redundant protein database limited to taxa *exovorus* and *qaytius*. These searches returned no significant similarity, except one alignment of Bd1029 against qaytius (97% query coverage, 46% amino acid identity). By comparison, our analysis of genome relatedness between HD100 and the two epibiotic predators estimated 59%−60% average amino acid identity (Fig. 2). Additionally, aligning Bd1029 to *Bacteriovorax* or *Halobacteriovorax* protein sequences returned full coverage alignments with 65%–69% identity. These BLAST results corroborate our conclusion that the Bd1029 and Bd2785 gene clusters found in all 16 intraperiplasmic predators in our data set have diverged substantially from the epibiotic lineage.

### Abundance and distribution of peptidoglycan-degrading and -modifying enzymes points to divergence and duplication of lineage-specific genes

To explore gene clusters’ predicted functions in more detail, we searched all annotation information associated with each gene cluster in the FULL pangenome for 17 terms describing enzymatic activities that can be important for predation (Table 3). This leveraged the combined output of multiple tools used to annotate protein sequences during pangenome construction. Our list of search terms includes key functions for bacterial predators, and we compiled it based on resources such as the initial annotation of *B. bacteriovorus* HD100 (2), recent studies of predator-specific enzyme functions (13), and Gene Ontology (23, 24). Some terms, such as hydrolase, peptidase, and nuclease, are broad and therefore associated with many gene clusters in the FULL pangenome; a substantial proportion of these gene clusters are singletons, occurring in only one chromosome of our data set (Table 3).

**TABLE 3:** Number of gene clusters in FULL pangenome predicted to encode listed enzymes^*a*^

	With singletons	Without singletons
Amidase^*b*^	152	70
Deacetylase^*b*^	172	76
Depolymerase	12	7
DNase	98	51
Endopeptidase^*b*^	216	98
Glucosaminidase	20	2
Glycanase	1	0
Glycosidase^*b*^	145	65
Hydrolase	4491	1997
Lipase	365	165
Lysozyme^*b*^	174	79
Muramidase^*b*^	10	7
Nuclease	1274	488
Peptidase	1957	909
Protease	996	492
RNase	190	100
Transglycosylase^*b*^	374	179

^*a*^ Because we compiled information from multiple annotation sources, a gene cluster can be associated with multiple search terms; therefore, we expect some overlap among categories, for example, peptidase and protease.

^*b*^ Enzymes chosen for additional analysis.

**Fig 6:**
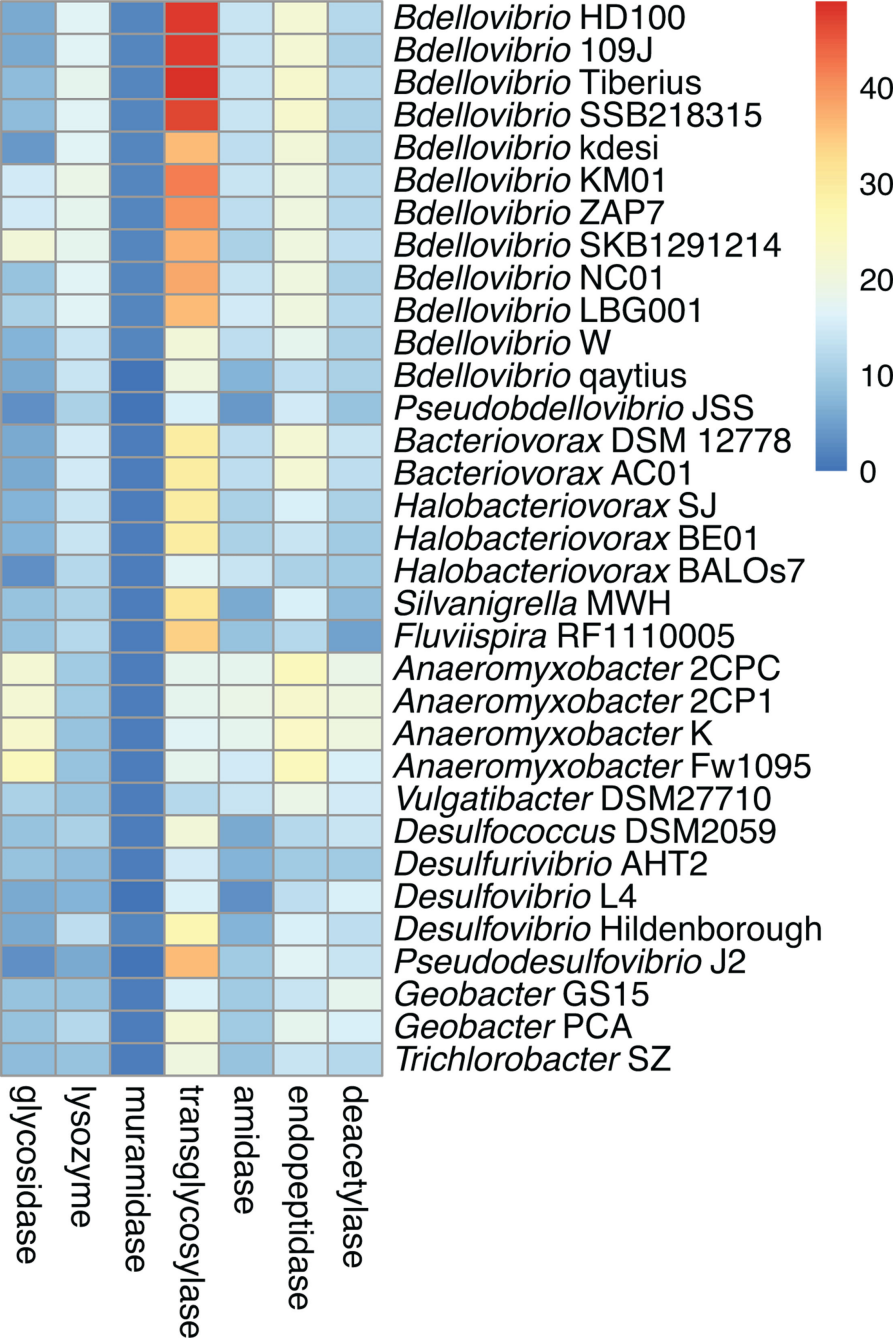
Abundance of gene clusters predicted to encode selected enzymes in each chromosome. For each gene cluster in the FULL pangenome, we searched all annotation information for seven enzyme terms. These enzymes define columns of the heatmap, and chromosomes define rows. The gradient scale reflects abundance of gene clusters; for example, *B. bacteriovorus* Tiberius has 49 gene clusters annotated as transglycosylase, and this number is represented by dark red in the heatmap. Some gene clusters are annotated with multiple terms; therefore, a gene cluster may be represented more than once in the heatmap.

We then narrowed our focus to seven terms: amidase, deacetylase, endopeptidase, glycosidase, lysozyme, muramidase, and transglycosylase (indicated with superscript in Table 3). These enzymes are active in multiple biological processes, including peptidoglycan metabolism. Of these seven terms, transglycosylase is associated with the largest number of gene clusters in the FULL pangenome (*n* = 374); just over half are singletons (Table 3). By contrast, very few gene clusters in the FULL pangenome are annotated as muramidase (*n* = 10). To compare abundances of the seven enzymes among the 33 bacteria in our data set, we built a heatmap showing the number of gene clusters predicted to encode these enzymes within each chromosome (Fig. 6). Gene clusters annotated as transglycosylase, endopeptidase, and lysozyme are highly abundant in intraperiplasmic *Bdellovibrio* compared to most other bacteria, with notably increased density of transglycosylase gene clusters in HD100 and closely related strains. Despite belonging to the same family as intraperiplasmic *Bdellovibrio*, the two epibiotic predators have fewer gene clusters predicted to encode each enzyme; in particular, JSS and qaytius have no gene clusters annotated as muramidase, no more than seven annotated as amidase, and no more than 15 annotated as endopeptidase.

**Fig 7:**
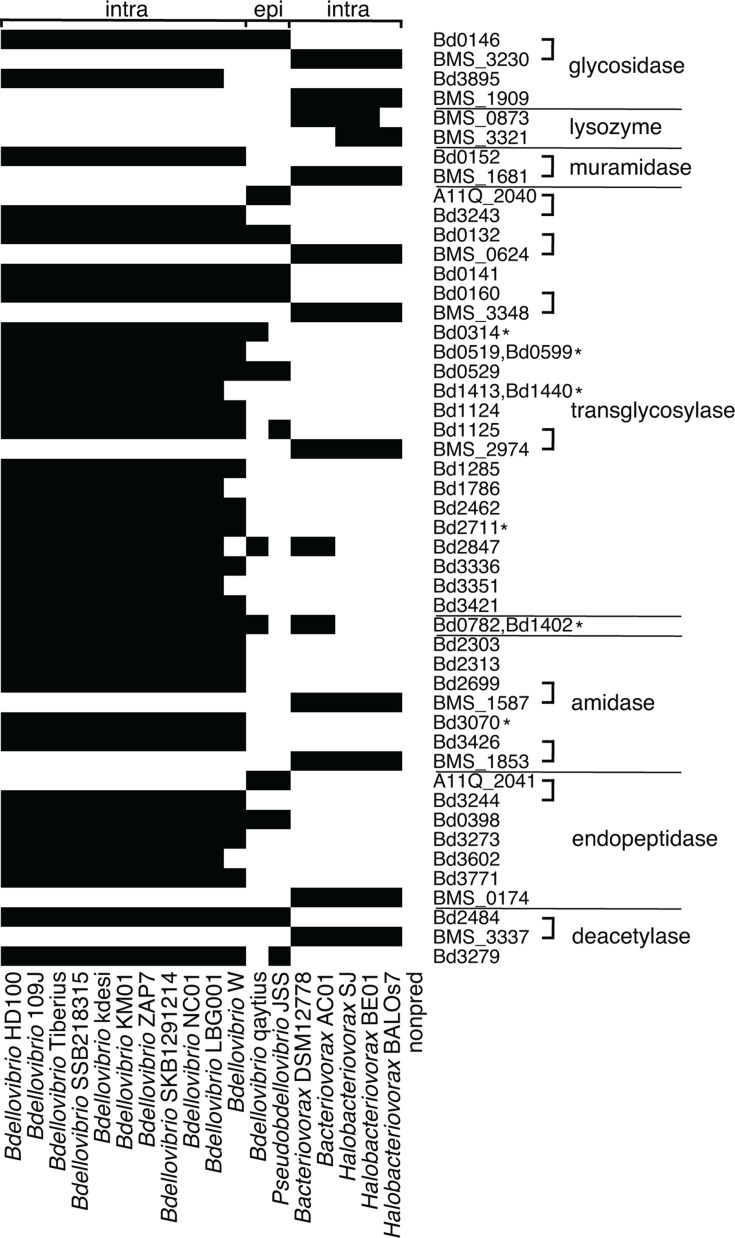
Distribution of lineage-specific enzyme gene clusters involved in peptidoglycan metabolism. Black indicates presence, and white indicates absence. Each row represents a unique gene cluster, identified by either *B. bacteriovorus* HD100 locus tag, *Halobacteriovorax marinus* SJ locus tag, or *Pseudobdellovibrio exovorus* JSS locus tag. Brackets indicate bidirectional best hits by TBLASTN. Asterisks indicate gene clusters that include multiple protein sequences from the same chromosome. If more than one HD100 protein sequence was assigned to a gene cluster, each HD100 gene’s locus tag is listed. Gene clusters are grouped by predicted function, using the terms assigned by PFAM and/or appearing most often in the combined output of the annotation tools.

To identify gene clusters that may have evolved in response to selective pressures associated with predation, we examined the distribution and predicted function of gene clusters annotated with any of the seven terms listed above. We first used presence–absence data to define a list of gene clusters broadly present in lineages of predatory bacteria in our data set and absent from all non-predatory bacteria. We then searched functional annotation information associated with these gene clusters in UniProt Knowledgebase. Because peptidoglycan metabolism, including modification and lysis of the prey cell wall, is very important for successful predation (25), we narrowed the list to gene clusters predicted or demonstrated to be involved in biosynthesis, degradation, and/or modification of peptidoglycan. This manual curation identified 48 lineage-specific enzyme gene clusters involved in peptidoglycan metabolism (Fig. 7). When one of these gene clusters was associated with more than one of the seven terms, we preferentially used the term assigned by PFAM and/or appearing most often in the combined output of the annotation tools.

Overall, distribution of the 48 lineage-specific enzyme gene clusters is largely restricted by family and predation strategy (Fig. 7). For example, the transglycosylase Bd1124 is found in all intraperiplasmic *Bdellovibrio* in our data set, but not found in the epibiotic predator genomes or *Bacteriovoracaceae* genomes. To explore the distribution of each gene cluster in Fig. 7, we used TBLASTN to align representative amino acid sequences from either HD100, JSS, or SJ against the six-frame translations of the genomes shown as lacking the gene cluster. We uploaded a summary file of the TBLASTN results to FigShare (https://doi.org/10.6084/m9.figshare.26828278.v1). Returning to Bd1124 as an example, TBLASTN yielded 24%–25% coverage and 35%–36% identity when aligning the HD100 Bd1124 protein sequence to epibiotic predator genomes and 23%–51% coverage and 26%–37% identity when aligning to *Bacteriovoracaceae* genomes. Given these short alignment lengths and our estimates of 51%–60% AAI when comparing the HD100 proteome to those of epibiotic predators and intraperiplasmic *Bacteriovoracaceae* (Fig. 2), our results indicate that the Bd1124 intraperiplasmic *Bdellovibrio* gene cluster has diverged substantially from the other predatory lineages and highlights it as a protein of interest for functional characterization.

Similar to Bd1124, many lineage-specific enzyme gene clusters shown in Fig. 7 aligned with <80% coverage and <40% identity to protein sequences from the other predatory lineages, which indicates substantial divergence and suggests these enzymes may be evolving in response to selective pressures related to predatory lifestyle. For some gene clusters in Fig. 7, the representative HD100 protein sequence and the representative JSS or SJ protein sequence were bidirectional best hits by TBLASTN, with 78%–93% coverage and 42%–50% identity for alignments of HD100 and JSS proteins and 71%–100% coverage and 29%–44% identity for alignments of HD100 and SJ proteins. When generating coarse gene clusters with the MCL algorithm, not all homologous sequences will cluster together if alignment length or sequence similarity is too low due to specific selective pressure on these genes and overall evolutionary distance among taxa. We indicated these bidirectional best hit gene cluster pairings with brackets in Fig. 7.

Within *Bdellovibrionaceae*, there are a substantial number of lineage-specific enzyme gene clusters conserved among the 11 intraperiplasmic *Bdellovibrio*; these gene clusters may constitute a core suite of peptidoglycan metabolism enzymes important for *Bdellovibrio* predation. Some of these gene clusters were not detected in kdesi and W; however, we note these chromosomes may not be fully finished (see Materials and Methods); therefore, this may be an artifact. Nineteen lineage-specific enzyme gene clusters found in intraperiplasmic *Bdellovibrio* are not shared with the two epibiotic predators in our data set (Fig. 7), which is consistent with the overall low abundance of gene clusters annotated with these enzymatic activities in JSS and qaytius. In particular, TBLASTN analysis returned “no significant similarity” when aligning HD100 protein sequences of amidases Bd0152 (specifically annotated as muramidase), Bd2313, and Bd3070 to six-frame translations of the epibiotic JSS and qaytius genomes. For each of these gene clusters, we identified homologous proteins in *Bacteriovoracaceae* by TBLASTN, which strongly indicates that an epibiotic predation strategy exerts different selective pressures on these amidase enzymes compared to intraperiplasmic predation.

Six lineage-specific enzyme gene clusters include multiple protein sequences from the same chromosome, providing evidence for recent gene duplication events in *Bdellovibrio* lineages (asterisks in Fig. 7, supplementary table on FigShare: https://doi.org/10.6084/m9.figshare.26778142.v2). Two gene clusters, each annotated as transglycosylase, include multiple protein sequences from a single chromosome, suggesting very recent gene duplication: LBG001 has four protein sequences assigned to the Bd0314 gene cluster, and SKB1291214 has two assigned to the Bd2711 gene cluster. The other four gene clusters include multiple protein sequences from multiple chromosomes. The Bd3070 amidase gene cluster includes two protein sequences from each of NC01, KM01, and ZAP7. A transglycosylase gene cluster includes two protein sequences from each of HD100 (Bd0519, Bd0599) and its three closest relatives 109J, Tiberius, and SSB218315, suggesting gene duplication in their ancestor. For another transglycosylase, most intraperiplasmic *Bdellovibrio* encode two protein sequences assigned to this gene cluster (for example, Bd1413 and Bd1440 from HD100), but KM01, ZAP7, and SKB1291214 only encode one, suggesting loss of the duplicated gene in their ancestor. Finally, a gene cluster annotated as L,D-transpeptidase includes multiple protein sequences from *Bdellovibrio* and *Bacteriovorax* genomes (for example, Bd0782 and Bd1402 from HD100).

## DISCUSSION

Since publication of the *Bdellovibrio bacteriovorus* HD100 genome 20 years ago (2), advances in sequencing technology and reductions in cost have opened the door for comparative genomics of predatory bacteria. Genome-wide analyses are an iterative process, with each study broadening and refining our knowledge of predatory bacteria genome evolution and variation as more predatory strains are isolated and sequenced. Here, we analyze the 18 obligate predatory bacteria genomes from *Bdellovibrionota* that were publicly available and defined as complete in the National Center for Biotechnology Information (NCBI) GenBank as of late December 2022 (Table 1). Our data set also includes complete genomes of 15 non-predatory strains from *Bdellovibrionota* and two related phyla. Using this data set, we investigated evolutionary relationships, genome relatedness, and genome content variation to better understand the diversity, distribution, and predicted function of protein families found in *Bdellovibrio* and its predatory relatives.

Phylogenetics of single-copy conserved genes combined with genome-wide analysis of average amino acid identity enabled us to reconstruct evolutionary relationships among the 33 bacteria in our data set and estimate genome relatedness among the 20 *Bdellovibrionota* strains, which includes 18 predators and two non-predators. The resulting phylogenetic tree (Fig. 1) and AAI heatmap (Fig. 2) highlight the importance of isolating and sequencing diverse predatory strains. For example, in addition to representing phenotypic variation in predation strategy, the two epibiotic strains JSS and qaytius represent valuable taxonomic and genomic diversity within the Family *Bdellovibrionaceae*, which includes intraperiplasmic *Bdellovibrio*. Our data support classifying JSS as a new genus, consistent with a recent proposal naming this genus *Pseudobdellovibrio* (10), and also suggest that qaytius represents a distinct novel genus. JSS has a reduced genome compared to intraperiplasmic *Bdellovibrio*, which led to a hypothesis that this epibiotic strain evolved from an intraperiplasmic ancestor via gene loss (26); however, completion of the qaytius genome, which is substantially larger than JSS, led to a hypothesis that epibiotic predation may have been the ancestral phenotype (9). Isolation, sequencing, and comparative analyses of additional epibiotic strains within *Bdellovibrionota* are essential to clarify evolutionary trajectories leading to different predation strategies.

Intraperiplasmic predator diversity is more well represented in our complete genome data set, constituting 16 of the 18 predatory bacteria genomes. Strains using this predation strategy are also more well-characterized in their molecular biology, with many studies of predatory phenotype and functional characterization of predatory proteins focused on HD100 or 109J. Our data underscore the high degree of genome relatedness between these two strains (Fig. 2) and demonstrate how recent isolation and sequencing of other *Bdellovibrio* strains expanded the diversity of complete genome information, with at least five distinct *Bdellovibrio* species suggested by phylogenetic position and AAI analysis. These strains and their genomes provide a useful tool for understanding the ecology and molecular biology of predatory bacteria and emphasize the ongoing need to sample the broad phenotypic and genomic space of predatory bacteria.

To investigate genome content variation among predatory bacteria and between predators and non-predators, we grouped predicted protein sequences from all 33 genomes by sequence similarity into “gene clusters,” which are a proxy for protein families. Our strategy for defining gene clusters aims to identify predatory bacteria proteins that have diverged substantially from homologous proteins in other lineages and thereby generate hypotheses about which proteins may encode functions important for predation. Very few gene clusters are conserved among all genomes (Table 2) or even all predatory bacteria genomes (Fig. 3). However, when we defined strain subsets based on taxonomy and predation strategy (for example, all intraperiplasmic *Bdellovibrio*), we identified gene clusters shared by strains in the subset and not found in any other strains (Fig. 4). Many of these “conserved and specific” gene clusters are predicted to function in “cell envelope biogenesis,” “signal transduction,” and “intracellular trafficking, secretion, and vesicular transport” (Fig. 5), which include processes important for predation. Our approach to identifying conserved and specific gene clusters highlights protein sequence divergence within broad functional categories; for example, considering gene clusters associated with cell envelope biogenesis (COG category M), there are some shared by intraperiplasmic and epibiotic *Bdellovibrionaceae*, but there are others found in only intraperiplasmic strains or only epibiotic strains. This suggests some proteins involved in cell envelope biogenesis may be experiencing greater sequence divergence due to selective pressures related to predation strategy. These proteins are strong candidates for experimental follow-up and functional characterization to better understand their roles in predation.

A substantial proportion of conserved and specific gene clusters identified for predatory bacteria lineages lack detailed functional annotation (Fig. 5), which underscores a gap in our knowledge. Experimental characterization of protein function can be difficult and time consuming. Annotation tools and protein family models offer computational alternatives; however, selective pressures associated with predatory lifestyle may result in molecular evolution and sequence divergence within some protein families that complicates sequence-based functional prediction. This may explain the low abundance of gene clusters assigned to selected enzymatic functions in the two epibiotic predators compared to intraperiplasmic predators (Fig. 6). With recent advances in protein structure prediction, new annotation approaches will combine sequence-based models with structural comparisons to predict protein function, which should provide greater insight into the roles and activities of predatory bacteria protein families (25).

For those gene clusters with predicted functions, we showed that intraperiplasmic *Bdellovibrio* are enriched for transglycosylases, endopeptidases, and lysozymes compared to non-predatory bacteria (Fig. 6). Because modification and lysis of the Gram-negative cell wall is such an important process in bacterial predation (25), we used annotation information to focus on gene clusters predicted to encode enzymes involved in peptidoglycan metabolism, including those that degrade and/or modify peptidoglycan. We identified 48 peptidoglycan metabolism gene clusters broadly found in predatory bacteria and not found in any non-predatory strains (Fig. 7). Experimental characterization of proteins belonging to these lineage-specific enzyme gene clusters is limited, but those with reported functions provide support that our approach can identify protein families with novel activities related to predation. For example, a deacetylase gene cluster found in all intraperiplasmic *Bdellovibrio* includes Bd3279, which is secreted into prey periplasm during the early stages of invasion and deacetylates prey peptidoglycan (13). A transglycosylase gene cluster also found in all intraperiplasmic *Bdellovibrio* includes Bd0314, which is described as a lysozyme with specificity for deacetylated peptidoglycan that plays an important role in exit of predatory progeny from the dead prey cell in the latter stages of the predatory lifecycle (12). Whereas only one HD100 protein sequence was assigned to this gene cluster, it includes four LBG001 protein sequences, suggesting recent gene duplication in this lineage and pointing to possible phenotypic variation in this strain.

Our independent identification of experimentally characterized predation-related enzymes via comparative genomics confirms the strength of this approach and argues for examining the suite of lineage-specific enzyme gene clusters listed in Fig. 7 for their effect on predatory phenotypes and possible novel activities. As more predatory bacteria genomes are completed and released, genome comparisons can guide functional characterization of proteins, inform hypotheses for experimental testing, and identify candidate enzymes for potential biotechnological and clinical applications.

## MATERIALS AND METHODS

### Data set of chromosome sequences for predatory bacteria and non-predatory close relatives

In December 2022, we used the NCBI Genome browser to search GenBank for publicly available genomes of predatory bacteria belonging to Phylum *Bdellovibrionota* that were listed as complete. This phylum includes the Family *Bdellovibrionaceae*, which encompasses intraperiplasmic and epibiotic predatory bacteria belonging to *Bdellovibrio* and *Pseudobdellovibrio*, and the Family *Bacteriovoracaceae*, which encompasses intraperiplasmic predatory bacteria belonging to *Bacteriovorax* and *Halobacteriovorax*. Our searches returned 18 genomes, and we included all 18 in our data set (Table 1). We also selected 15 genomes from non-predatory bacteria belonging to *Bdellovibrionota* (two strains), *Myxococcota* (five strains), and *Desulfobacterota* (eight strains) (Table 1). We conducted literature searches to confirm these strains are not obligate predators; we acknowledge it is possible one or more of these strains have undiscovered predatory capabilities.

While curating our data set, we noted the chromosomes for kdesi and W are smaller than average for *Bdellovibrio* strains. The GenBank genome record for kdesi reports assembly as reference-guided alignment of Illumina MiSeq reads against HD100, and CheckM analysis shows 89% completeness, suggesting the current version of the kdesi genome may be missing sequence. The genome sequence for W was recently excluded from RefSeq by NCBI staff because genome length was deemed too small (defined by NCBI as “total non-gapped sequence length of the assembly is less than half that of the average for the genomes in the Assembly resource from the same species…or is otherwise suspiciously short”). Tudor and McCann discussed preliminary sequencing and analysis of W in Reference (22); however, they stated genome assembly was not complete, and the genome sequence was not closed. We were unable to find a later reference reporting details of sequencing and assembly, and the GenBank nucleotide record does not include this information. We elected to include both kdesi and W in our data set because their positions on the phylogenetic tree demonstrate that they represent valuable strain diversity.

After finalizing our data set, we downloaded FASTA and GBFF files for each genome from NCBI Genomes in late December 2022. GBFF files include nucleotide sequence and annotation information from NCBI’s Prokaryotic Genome Annotation Pipeline (PGAP) (27). Because our analyses focus on chromosome sequences, we removed plasmid sequences from seven records (*Halobacteriovorax marinus* SJ, *Silvanigrella aquatica* MWH-Nonnen-W8red, *Fluviispira sanaruensis* RF1110005, *Geobacter metallireducens* GS-15, *Trichlorobacter lovleyi* SZ, *Desulfovibrio vulgaris* Hildenborough, *Desulfovibrio desulfuricans* L4). We then screened all 33 chromosome sequences for International Union of Pure and Applied Chemistry (IUPAC) ambiguity codes. *Pseudobdellovibrio exovorus* JSS used these codes at 52 nucleotide positions, and we converted each position to N to indicate a nucleotide is present, but its identity is not known.

### Building pangenomes of chromosome sequences with anvi’o

We processed and analyzed chromosome sequences using anvi’o version 7.1 (17, 18). Briefly, for each chromosome sequence, we provided the GBFF file as input and built a contigs database, using NCBI gene calls and invoking skip-predict-frame for all records except *Silvanigrella*, which had partial gene calls that required translation frame prediction by anvi’o. To compile functional annotation information for each chromosome, we first used anvi-get-sequences-for-gene-calls to generate a proteome file from the contigs database containing all predicted amino acid sequences. We then analyzed this file with eggNOG mapper and InterProScan. Annotation with eggNOG mapper was performed using emapper-2.1.6 (28) based on eggNOG 5.0 orthology data (29), with sequence searches performed using DIAMOND 2.0.11 (30). Annotation with InterProScan used version 5.60–92.0 (31, 32) and invoked the following: FunFam-4.3.0 (33), PANTHER-17.0 (34), Pfam-35.0 (35), ProSitePatterns-2022_01 and ProSiteProfiles-2022_01 (36), SUPERFAMILY-1.75 (37), and TIGRFAM-15.0 (38). Using anvi-import-functions, we added annotation information to each contig database from NCBI PGAP (created during initial processing), eggNOG mapper, and InterProScan. We also added annotation information from anvi’o default HMM databases using anvi-run-hmms.

After processing chromosome sequences and adding annotation information, we followed the workflow described at https://merenlab.org/2016/11/08/pangenomics-v2/ (last accessed 20 February 2023) to build pangenomes. We defined three pangenomes: FULL, consisting of all 33 predatory and non-predatory bacteria; PRED, consisting of 18 predatory bacteria; and BDELLO, consisting of 13 predatory bacteria belonging to the Family *Bdellovibrionaceae*. For each chromosome within a pangenome, anvi’o translated open reading frames predicted by PGAP, then clustered amino acid sequences based on sequence similarity using BLASTP (39) and MCL (19, 40, 41). Following anvi’o terminology, we refer to these as “gene clusters.” Based on recommendations in the MCL algorithm manual at https://micans.org/mcl/man/mcl.html (last accessed on 1 March 2023), we tested three different MCL inflation parameters: 1.4, 2, and 6. After evaluating the effect of these values (Table 2), we chose 1.4 to generate “coarse” gene clusters because of the large phylogenetic distance among genomes in our data set. After setting this value, we built two versions of the pangenome for FULL, PRED, and BDELLO: one with singletons (default settings) and one without singletons (default settings except --min-occurrence 2). We uploaded a summary file to FigShare with information on every gene cluster in the FULL pangenome without singletons (https://doi.org/10.6084/m9.figshare.26778130.v1).

### Constructing a single-copy core gene cluster phylogenetic tree with IQTree

We adapted the workflow described at https://merenlab.org/data/spiroplasma-pangenome/ (last accessed 15 March 2023) to build a phylogenetic tree. Within anvi’o, we defined single-copy core gene clusters for the FULL pangenome using anvi-get-sequences-for-gene-clusters with “min-num-genomes-gene-cluster-occurs 33” to ensure representation in all 33 chromosomes, “max-num-genes-from-each-genome 1” to ensure occurrence as only a single copy, “max-functional-homogeneity-index 0.95” to exclude gene clusters of identical or nearly identical amino acid sequences because they are not phylogenetically informative, and “min-geometric-homogeneity index 0.8.” These parameter values yielded 44 single-copy core gene clusters containing 1,452 amino acid sequences. As output, anvi’o generated an alignment of the concatenated amino acid sequences from each chromosome.

We trimmed the alignment using trimAl v1.4.rev15 (42) with the setting automated1, which is optimized for Maximum Likelihood phylogenetic tree reconstruction. Using IQtree v2.2.0.3 (43), we constructed a phylogenetic tree using the LG + F + I + I + R5 model, which was selected by ModelFinder (44) as the best-fit model. Bootstrap values were estimated by IQtree with ultrafast bootstrap approximation (45) using 1,000 samples.

### Determining average amino acid identity (AAI) with EzAAI

To calculate AAI, we used EzAAI v1.2.2 (46) with the proteome files generated by anvi-get-sequences-for-gene-calls during the pangenome workflow described above. For each possible pairwise comparison among the 33 strains of our data set, EzAAI implemented MMSeqs2 (47) to align amino acid sequences and identify bidirectional best hits with at least 25% identity and at least 50% coverage. A bidirectional best hit, also referred to as a reciprocal hit, is defined as a pair of amino acid sequences from two different genomes for which each sequence is the other’s best match across the entire proteome. Using all bidirectional best hits from a given pair of chromosomes, EzAAI then calculated an overall average amino acid identity (AAI) for that pair. EzAAI also outputs proteome coverage, which is defined as the proportion of the combined proteome of a pair of genomes that is comprised of best bidirectional hits. Proteome coverage decreases with increasing phylogenetic distance (46); therefore, this index provides a measure of how much information was used to calculate AAI. We generated a heatmap of AAI and proteome coverage data in R, using the ComplexHeatMap package v2.15.1 (48) and the circlize package v0.4.15 (49).

### Analyzing gene cluster distribution

For the FULL pangenome without singletons, we used anvi-compute-functional-enrichment-in-pan to generate a presence–absence matrix for each gene cluster. This matrix lists gene clusters by their unique GC ids and shows how many amino acid sequences from each chromosome were assigned to each gene cluster. To analyze gene cluster distribution, we classified strains by taxonomy and predation strategy into four sets as follows: intraperiplasmic *Bdellovibrionaceae* (Bdello_intra), epibiotic *Bdellovibrionaceae* (Bdello_epi), *Bacteriovoracaceae*, and non-predatory strains (nonpred). Bdello_intra includes 11 *Bdellovibrio* strains known to invade the periplasm of Gram-negative prey. Bdello_epi consists of *Pseudobdellovibrio exovorus* JSS and *Ca.* Bdellovibrio qaytius, which attach to the surface of prey bacteria but do not invade. *Bacteriovoracaceae* is represented by five *Bacteriovorax* and *Halobacteriovorax* strains, which are known to invade the periplasm of Gram-negative prey but are phylogenetically distinct from *Bdellovibrio*. Nonpred includes the remaining 15 strains in our data set, which are non-predatory strains from *Bdellovibrionota* and two other phyla that were previously grouped together in *Deltaproteobacteria*.

After defining these sets, we used the R packages dplyr (50), tidyr (51), tools (52), and UpSetR (53) to identify gene clusters conserved within a single set or among a group of sets but entirely absent from the other sets. In other words, we wanted to find gene clusters present in every chromosome of a particular combination of strains but not detected in any of the other strains’ chromosomes. We retrieved functional annotations and COG classifications for these gene clusters from the FULL pangenome.

### Analyzing lytic enzyme gene cluster distribution

We used anvi-search-functions to search protein sequence functional annotations in the FULL pangenome for the 17 terms listed in Table 3. This generated counts of gene clusters associated with each term. Some gene clusters are annotated with more than one term and therefore are counted more than once in Table 3. We further investigated seven lytic enzymes as follows: amidase, deacetylase, endopeptidase, glycosidase, lysozyme, muramidase, and transglycosylase. For gene clusters associated with these enzymatic activities, we retrieved presence–absence data from the FULL pangenome without singletons and analyzed the data for gene clusters unique to predatory bacteria. In some cases, these gene clusters were annotated with more than one of the seven terms. We resolved this by examining annotation information and selecting the term assigned by PFAM annotation and/or occurring most often. We used the pheatmap package in R (54) to explore distribution of these gene clusters and identify lineage-specific proteins, defined as gene clusters broadly present in groups of predatory bacteria but never found in non-predatory bacteria. To narrow this list of lineage-specific enzymes to those involved in peptidoglycan metabolism, we searched for gene name (when assigned) or locus tag in the UniProt Knowledgebase (uniprot.org), which compiles annotation information from multiple sources. Gene clusters with predicted functions related to peptidoglycan biosynthesis, modification, or degradation are shown in the heatmap presented in Fig. 7.
